# Effect of Weight Change on Type 2 Diabetes Risk: A Prospective Study in a Japanese Population

**DOI:** 10.1210/clinem/dgaf558

**Published:** 2025-10-08

**Authors:** Takuya Kagisaki, Megu Y Baden, Asuka Oyama, Midori Noguchi, Hiroyasu Iso, Iichiro Shimomura

**Affiliations:** Department of Metabolic Medicine, Graduate School of Medicine, The University of Osaka, Suita, Osaka 565-0871, Japan; Department of Metabolic Medicine, Graduate School of Medicine, The University of Osaka, Suita, Osaka 565-0871, Japan; Department of Lifestyle Medicine, Graduate School of Medicine, The University of Osaka, Suita, Osaka 565-0871, Japan; Health and Counseling Center, The University of Osaka, Toyonaka, Osaka 560-0043, Japan; Department of Public Health, Graduate School of Medicine, The University of Osaka, Suita, Osaka 565-0871, Japan; Institute for Global Health Policy Research, Bureau of Global Health Cooperation, Japan Institute for Health Security, Shinjuku, Tokyo 162-8655, Japan; Department of Metabolic Medicine, Graduate School of Medicine, The University of Osaka, Suita, Osaka 565-0871, Japan

**Keywords:** prospective cohort study, epidemiology, type 2 diabetes, weight change, diabetes prevention

## Abstract

**Context:**

Although weight reduction has been shown to reduce type 2 diabetes risk, the threshold of weight change, including increase and decrease, associated with the risk remained unclear.

**Objective:**

We investigated the association between weight change and the risk of type 2 diabetes.

**Methods:**

The participants were 41 539 nondiabetic residents (16 914 men, 24 625 women) in Kochi, Japan, who underwent health examinations between April 2013 and March 2015. The association between 1-year weight change and the risk of type 2 diabetes was assessed using Cox proportional hazards models.

**Results:**

During a mean 5.7-year follow-up, 3564 new-onset type 2 diabetes cases were documented. Compared with participants with stable body weight (<1.0% change), those with weight decreases of 1.0% to 2.9%, 3.0% to 4.9%, and 5.0% or greater showed a lower risk of type 2 diabetes (16% [8%-24%; *P* < .001]; 25% [15%-34%; *P* < .001]; and 26% [14%-36%; *P* < .001], respectively). Conversely, those with weight increases of 1.0% to 2.9%, 3.0% to 4.9%, and 5.0% or greater showed a higher risk (15% [4%-26%; *P* = .005], 35% [20%-53%; *P* < .001], and 51% [29%-76%; *P* < .001], respectively). These associations persisted among individuals with a body mass index (BMI) of 25 or greater or a 10-kg or greater weight gain since age 20 years. Among individuals with BMI 22 to 25, a weight increase of 3.0% or greater showed a higher risk. No associations were observed in individuals with a BMI of less than 22 or aged 75 or older.

**Conclusion:**

Both preventing weight gain and reducing weight are important for lowering the risk of type 2 diabetes in Japanese individuals, particularly among those with a BMI of 22 or greater.

Recently, type 2 diabetes has been increasing globally in line with increasing obesity (1). Based on previous evidence linking weight reduction to lower risk type 2 diabetes risk, several guidelines have recommended reducing weight to prevent type 2 diabetes. For example, the American Diabetes Association recommends a 3% to 7% weight reduction for individuals with overweight or obesity to prevent type 2 diabetes (2-7). Similarly, the Japan Society for the Study of Obesity recommends a 3% weight reduction over a 3- to 6-month period for people with obesity (8, 9). In addition, the Japan Diabetes Society recommends a 2-kg reduction in body weight to prevent the onset of type 2 diabetes (10-13).

Although many studies have investigated the effect of weight reduction on glycemic control among people with overweight, obesity, and glucose intolerance, few studies have examined the relationship between weight gain and the risk of type 2 diabetes, particularly among individuals without obesity (14). In addition, most previous studies on type 2 diabetes risk have defined the disease onset solely based on laboratory data or disease classification codes. This approach may lead to misclassification, as disease classification codes can be assigned to nondiabetic individuals who visit medical institutions for diabetes screening, and the type of diabetes cannot be determined based solely on laboratory data at health examinations. Furthermore, as epidemiological studies revealed that type 2 diabetes in Asia is characterized by a relatively low body mass index (BMI) onset (15), it is important to reveal the association between weight change and the risk of type 2 diabetes in individuals without obesity when considering the Asian population.

Therefore, in this study, we investigated the association between weight change and the risk of developing type 2 diabetes in people without diabetes, including those without obesity. By leveraging a large medical database in Japan, namely, the Kokuho Database (KDB), which includes health examination data, disease classification codes, and prescription records from monthly health insurance claims, we were able to more accurately define both the onset and classification of type 2 diabetes.

## Materials and Methods

### Study Population and Design

The present study is a prospective cohort study using the medical database of residents in Kochi Prefecture, Japan, maintained by the Federation of National Health Insurance Organizations. In Japan, individuals enrolled in the National Health Insurance and the medical care system for older individuals in the latter stage of life, which are available to those aged 40 to 74 and 75 and older, respectively, are recommended to undergo annual health examinations. The KDB contains both health examination data and monthly health insurance claims data, including records of prescriptions, medical treatments, and disease classification codes, for the National Health Insurance and the medical care system for older individuals in the latter stage of life.

**Figure 1.**
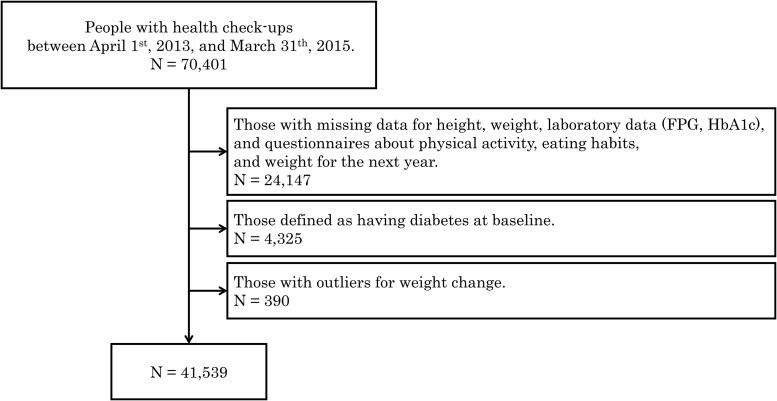
Flowchart of the selection of study participants.

In this study, baseline was defined as the first health examination attended between 2013 and 2014 (April 1, 2013, to March 31, 2015). A total of 70 401 participants were included and followed until 2021. We excluded participants with missing data on height, weight, laboratory data on fasting plasma glucose and glycated hemoglobin A_1c_ (HbA_1c_) levels, physical activity, dietary habits, and those with missing data at 1 year later. Additionally, participants who had diabetes at their baseline examination, and those who exhibited outliers for weight change, defined as being outside the range of the mean ± 3 SDs, were excluded. After exclusions, 41 539 participants were included in our analysis (Fig. 1). This study was approved by the institutional ethics review board of the University of Osaka Hospital (approval No. 20209).

### Definition of Type 2 Diabetes

The onset of diabetes was defined as an HbA_1c_ of 6.5% or greater, fasting plasma glucose levels of 126 mg/dL or greater, or first prescription for antihyperglycemic medication. Additionally, we used prescriptions records and the use of blood glucose self-monitoring devices to define type 2 diabetes accurately.

**Figure 2.**
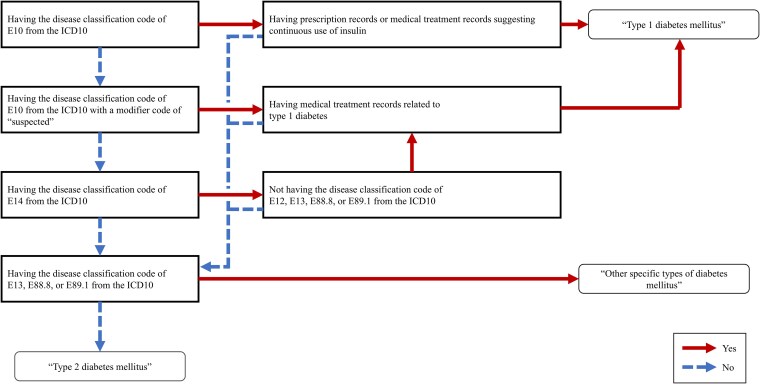
Flowchart for the definition of diabetes in this study.

Type 2 diabetes was defined as diabetes that was not classified as type 1 diabetes or other specific types of diabetes (Fig. 2). Type 1 diabetes was defined when one of the following 3 criteria was met: (a) the disease classification code was E10 (type 1 diabetes mellitus) in the International Statistical Classification of Diseases and Related Health Problems (ICD10), and prescription records or medical treatment records indicated continuous use of insulin; (b) the disease classification code was E10 (type 1 diabetes mellitus) in the ICD10 with a modifier code of “suspected,” the prescription records indicated continuous use of insulin, and records of the use of blood glucose self-monitoring devices related to type 1 diabetes were present; or (c) the disease classification code was E14 (unspecified diabetes mellitus) in the ICD10, there were no disease classification codes related to other types of diabetes mellitus, the prescription records indicated continuous insulin use, and records of the use of blood glucose self-monitoring devices related to type 1 diabetes were present. The disease classification defined other specific types of diabetes as follows: E13 (other specified diabetes mellitus), E88.8 (other specified metabolic disorder), and E89.1 (postprocedural hypoinsulinemia).

### Exposure Assessment

We assessed the weight change over 1 year by calculating the percentage of weight that each participant had gained or lost in the subsequent year (weight change [%] = (weight at next year [kg] − weight at baseline [kg])/weight at baseline [kg] × 100).

### Assessment of Covariates

Data on age and sex were obtained from the list of subscribers included in the KDB. BMI was calculated using the data on height and weight at baseline, where weight (kg) is divided by height squared (m^2^). Information on physical activity, walking speed, and dietary habits was collected by using data from the self-administered questionnaire developed by the Ministry of Health, Labour and Welfare of Japan (16). Physical activity was assessed by 2 questions as follows: “Are you in the habit of doing exercise to sweat lightly for over 30 minutes a time, 2 times weekly, for over a year? (yes or no)” and “In your daily life do you walk or do any equivalent amount of physical activity for more than one hour a day? (yes or no).” Participants who answered “no” to both of these questions were defined as having less physical activity. Walking speed was assessed with the following question: “Is your walking speed faster than that of people of your age and sex?” (yes or no). Eating habits were assessed by using the following questions: “Is your eating speed quicker than others? (quicker, normal, or slower)”; “Do you eat supper two hours before bedtime more than 3 times a week? (yes or no)”; “Do you eat snacks after supper more than 3 times a week? (yes or no)”; “Do you skip breakfast more than 3 times a week? (yes or no)”.

### Statistical Analyses

The Cox proportional hazards model was applied to examine the association between weight change and the risk of type 2 diabetes. The exposure variable was weight change, which was divided into 7 subgroups (≤−5.0%, > −5.0% and ≤ −3.0%, > −3.0% and ≤ −1.0%, > −1.0% and <+1.0%, ≥+1.0% and <+3.0%, ≥+3.0% and <+5.0%, and ≥+5.0%). Covariates were age, sex, BMI, HbA_1c_, physical activity, walking speed, and the number of poor eating habits (eating fast, skipping breakfast, eating dinner late, and snacking after dinner).

As sensitivity analyses, we stratified participants by sex, HbA_1c_, BMI, waist circumference, weight change from age 20, and age. The cutoff for HbA_1c_ was based on the definition of prediabetes in the American Diabetes Association, specifically an HbA_1c_ of 5.7% or greater (17). The BMI cutoffs were based on the average value of BMI associated with low mortality in the Japanese population (BMI = 22) (18) and obesity (BMI ≥ 25) established by the Japan Society for the Study of Obesity (8). The waist circumference cutoff was based on the criteria for metabolic syndrome in Japan, specifically 85 cm or greater for men and 90 cm or greater for women (8). Weight change from age 20 was assessed with the following question: “Have you gained over 10 kg from your weight at age 20?” The age cutoffs were based on the definition of former-stage older adults (aged 65-74 years) and latter-stage older adults (aged ≥75 years), as outlined in the Act on Assurance of Medical Care for Elderly People in Japan (19).

All analyses were performed using Python version 3.9.18 (Python Software Foundation) and the lifelines package version 0.27.8 (20). The statistical significance level was set at a 2-sided *P* less than .05.

## Results

### Participant Characteristics

During the mean follow-up period of 5.7 years, we documented 3564 cases of new-onset type 2 diabetes, yielding a crude incidence rate of 15.0 per 1000 person-years. Table 1 shows the baseline characteristics of the participants. The mean age of the participants was 64.6 years, 40.7% of the participants were men, and the mean BMI was 22.9.

**Table 1.** Baseline characteristics

No.	41 539
Age, y	64.6 (8.3)
Sex, men/women, %	40.7/59.3
Height, cm	157.3 (8.7)
Weight, kg	56.8 (10.7)
BMI	22.9 (3.2)
Systolic blood pressure, mm Hg	128.0 (17.8)
Diastolic blood pressure, mm Hg	75.2 (10.8)
HDL-C, mg/dL	60.7 (15.8)
LDL-C, mg/dL	120.7 (29.8)
Triglycerides, mg/dL	122.9 (79.4)
HbA_1c_ (NGSP), %	5.6 (0.3)
Fast eating, %	27.8
Skipping breakfast, %	6.8
Late dinner, %	16.6
Snack after dinner, %	13.3
Less physical activity, %	33.0
Walking fast, %	50.4

Data are the mean (SD) or percentage.

Abbreviations: BMI, body mass index; HbA_1c_, glycated hemoglobin A_1c_; HDL-C, high-density lipoprotein cholesterol; LDL-C, low-density lipoprotein cholesterol; NGSP, national glycohemoglobin standardization program.

### Changes in Body Weight and Risk of Type 2 Diabetes

**Figure 3.**
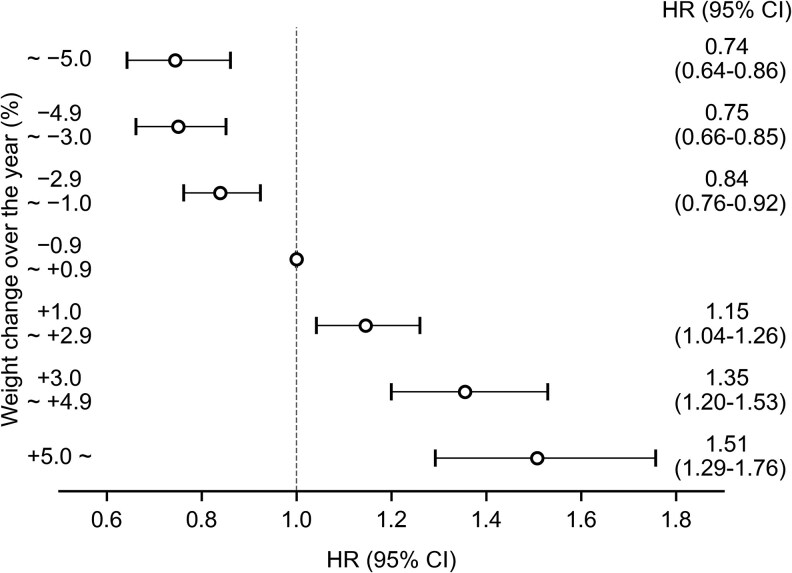
Association between weight change and the risk of type 2 diabetes. Adjusted for age, sex, body mass index, glycated hemoglobin A_1c_, physical activity, walking speed, and eating habits. Abbreviation: HR, hazard ratio.

After adjusting for age, sex, BMI, HbA_1c_, physical activity, walking speed, and eating habits, compared with the participants with no change in body weight (−0.9% to 0.9%), those with a weight decrease of 1.0% to 2.9%, 3.0% to 4.9%, and 5.0% or more were associated with a lower hazard ratio (HR) for type 2 diabetes (0.84 [0.76-0.92]; *P* < .001; 0.75 [0.66-0.85]; *P* < .001; and 0.74 [0.64-0.86]; *P* < .001; respectively) (Fig. 3).

In contrast, compared with participants with no change, those with a weight increase of 1.0% to 2.9%, 3.0% to 4.9%, and 5.0% or more exhibited a higher HR for type 2 diabetes (1.15 [1.04-1.26]; *P* = .005; 1.35 [1.20-1.53]; *P* < .001; and 1.51 [1.29-1.76]; *P* < .001; respectively) (see Fig. 3). On examining the trend, a statistically significant positive relationship was found between weight change and the risk of type 2 diabetes (*P* trend < .001; see Fig. 3).

### Associations Between Body Weight and Risk of Type 2 Diabetes Stratified by Body Mass Index, Waist Circumference, and Weight Change From Age 20

**Figure 4.**
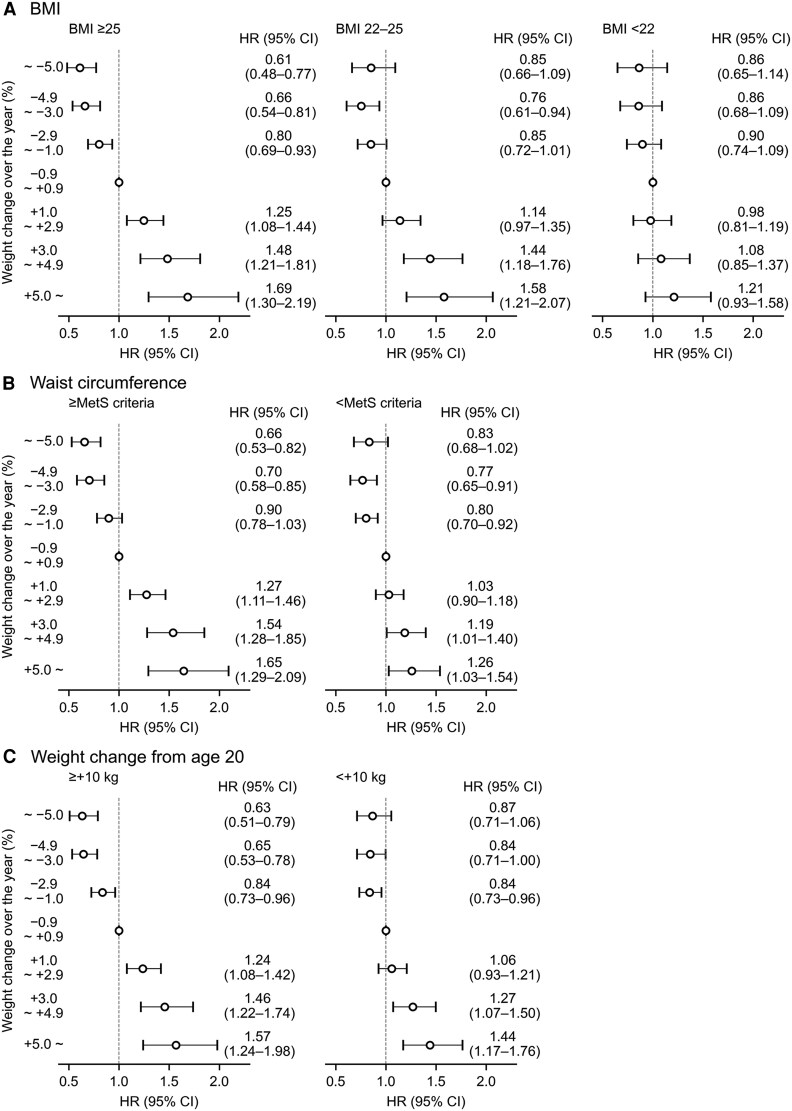
Stratified analyses of the association between weight change and the risk of type 2 diabetes by A, BMI; B, waist circumference; and C, weight change from age 20 years. A and B, Adjusted for age, sex, HbA_1c_, physical activity, walking speed, and eating habits. C, Adjusted for age, sex, BMI, HbA_1c_, physical activity, walking speed, and eating habits. Metabolic syndrome criteria: 85 cm or greater for men and 90 cm or greater for women. Abbreviations: BMI, body mass index; HbA_1c_, glycated hemoglobin A_1c_; HR, hazard ratio.

When participants were stratified into 3 BMI groups (<22, 22-25, and ≥25), we observed positive associations between weight change and type 2 diabetes risk in those with a BMI of 22 to 25 and those with a BMI of 25 or greater (*P* trends < .001) (Fig. 4A). Among participants with a BMI of 22 to 25, weight increases of 3.0% to 4.9% and 5.0% or more were associated with higher risks of 44% (18%-76%) and 58% (21%-107%), respectively, whereas a weight decrease of 3.0% to 4.9% was associated with a lower risk of 24% (6%-39%). Among participants with a BMI of 25 or greater, weight increases of 1.0% to 2.9%, 3.0% to 4.9%, and 5.0% or greater were associated with higher risks of 25% (8%-44%), 48% (21%-81%), and 69% (30%-119%), respectively, whereas weight decreases of 1.0% to 2.9%, 3.0% to 4.9%, and 5.0% or greater were associated with lower risks of 20% (7%-31%), 34% (19%-46%), and 39% (23%-52%), respectively. In contrast, among participants with a BMI of less than 22, no associations were observed between each category of weight change and the risk of type 2 diabetes, although an overall trend was observed (*P* for trend < .01).

Similar associations were shown when participants were stratified based on whether their waist circumference met the criteria for metabolic syndrome or by whether they had gained more than 10 kg since age 20 years (Fig. 4B and 4C). For analyses on waist circumference, data from 40 330 participants with available measurements were used (waist circumference data were missing for 1209 individuals). Among those whose waist circumference met the criteria for metabolic syndrome, weight increases of 1.0% to 2.9%, 3.0% to 4.9%, and 5.0% or more were associated with 27% (11%-46%), 54% (28%-85%), and 65% (29%-109%) higher risks, respectively, whereas weight decreases of 3.0% to 4.9% and 5.0% or greater were associated with 30% (15%-42%) and 34% (18%-47%) lower risks (see Fig. 4B). Among participants whose waist circumference did not meet the criteria for metabolic syndrome, weight increases of 3.0% to 4.9% and 5.0% or greater were associated with 19% (1%-40%) and 26% (3%-54%) higher risks, whereas weight decreases of 1.0% to 2.9% and 3.0% to 4.9% were associated with 20% (8%-30%) and 23% (9%-35%) lower risks.

Similarly, among participants who gained more than 10 kg since age 20, weight increases of 1.0% to 2.9%, 3.0% to 4.9%, and 5.0% or greater were associated with higher risks of type 2 diabetes (24% [8%-42%], 46% [22%-74%], and 57% [24%-98%], respectively), and weight decreases of 1.0% to 2.9%, 3.0% to 4.9%, and 5.0% or greater in this group were associated with lower risks (16% [4%-27%], 35% [22%-47%], 37% [21%-49%], respectively) (see Fig. 4C). Among those who did not gain more than 10 kg since age 20, weight increases of 3.0% to 4.9%, and 5.0% or greater were associated with higher risks of type 2 diabetes (27% [7%-50%], 44% [17%-76%]), whereas a weight decrease of 1.0% to 2.9% was associated with a lower risk (16% [4%-27%]).

### Associations Between Body Weight and Risk of Type 2 Diabetes Stratified by Age

**Figure 5.**
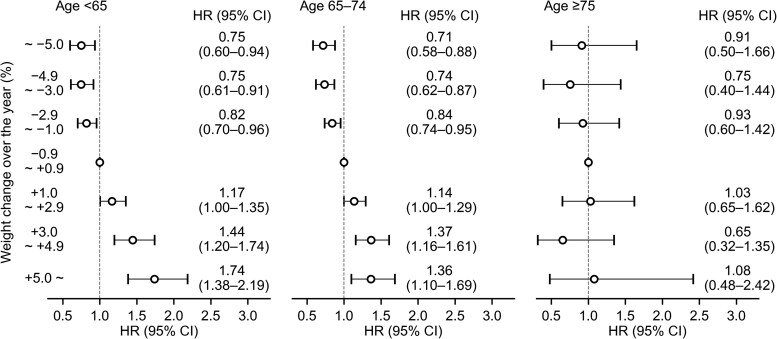
Association between weight change and the risk of type 2 diabetes stratified by age group (<65 years, 65-74 years, and ≥75 years). Adjusted for sex, body mass index, glycated hemoglobin A_1c_, physical activity, walking speed, and eating habits. Abbreviation: HR, hazard ratio.

Furthermore, we stratified participants into 3 groups by age (<65 years, 65-74 years, and ≥75 years) at baseline (Fig. 5). We observed positive associations between weight change and the risk of type 2 diabetes among those younger than 65 years and those aged 65 to 74 years (both *P* trends < .001). Among those younger than 65, weight increases of 3.0% to 4.9% and 5.0% or greater were associated with 44% (20%-74%) and 74% (38%-119%) higher risks, and weight decreases of 1.0% to 2.9%, 3.0% to 4.9%, and 5.0% or greater were associated with 18% (4%-30%), 25% (9%-39%), and 25% (6%-40%) lower risks. Among those aged 65 to 74, weight increases of 3.0% to 4.9% and 5.0% or greater were associated with 37% (16%-61%) and 36% (10%-69%) higher risks, respectively, whereas weight decreases of 1.0% to 2.9%, 3.0% to 4.9%, and 5.0% or greater were associated with 16% (5%-26%), 26% (13%-38%), and 29% (12%-42%) lower risks. On the contrary, there was no association between weight change and risk of type 2 diabetes among participants aged 75 years or older (*P* trend = .78).

### Associations Between Body Weight and Risk of Type 2 Diabetes Stratified by Glycated Hemoglobin A_1c_ and Sex

**Figure 6.**
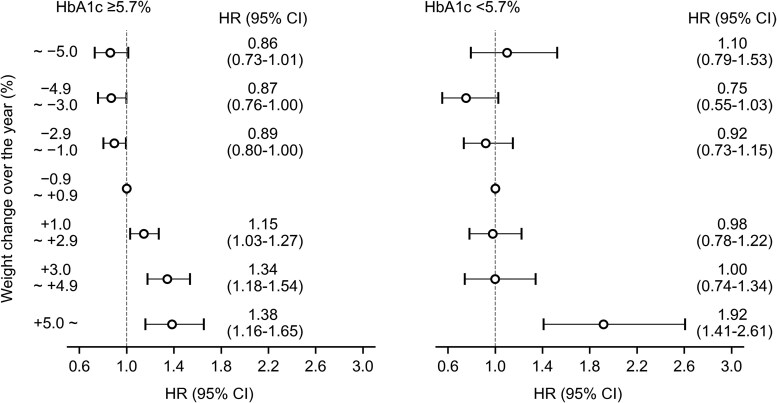
Association between weight change and the risk of type 2 diabetes stratified by glycated hemoglobin A_1c_ at baseline. Adjusted for age, sex, body mass index, physical activity, walking speed, and eating habits. Abbreviation: HR, hazard ratio.

In addition, we stratified participants into 2 groups by baseline HbA_1c_. Among participants with an HbA_1c_ of 5.7% or greater, weight increases of 1.0% to 2.9%, 3.0% to 4.9%, and 5.0% or greater were associated with higher risks of type 2 diabetes (15% [3%-27%], 34% [18%-54%], and 38% [16%-65%], respectively). Among those with an HbA_1c_ of less than 5.7%, a weight increase of 5.0% or greater was associated with a higher risk of type 2 diabetes (92% [41%-161%]) (Fig. 6).

**Figure 7.**
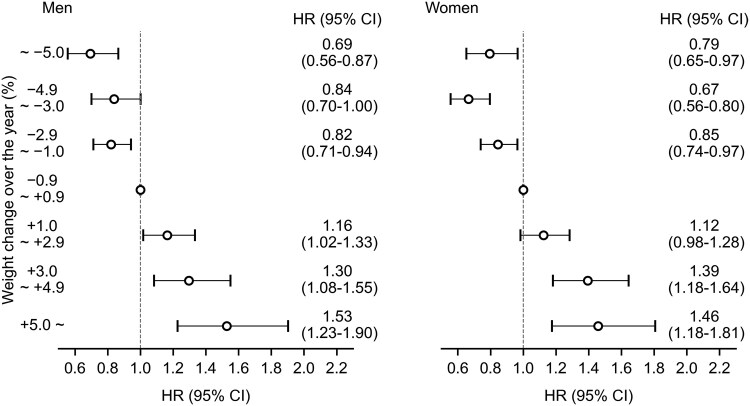
Association between weight change and the risk of type 2 diabetes stratified by sex. Adjusted for age, body mass index, glycated hemoglobin A_1c_, physical activity, walking speed, and eating habits. Abbreviation: HR, hazard ratio.

When we stratified participants by sex, no apparent difference was observed between men and women in the association between weight change and risk of type 2 diabetes, with positive associations observed both in men and women (Fig. 7).

## Discussion

In this study, we investigated the association between weight change and the risk of type 2 diabetes by using the KDB medical database. Participants with a weight increase of 1% or more per year were associated with a higher risk of type 2 diabetes compared with those without weight changes, independent of age, sex, HbA_1c_, BMI, physical activity, walking speed, and eating habits. In contrast, a weight decrease of 1% or more over the year was associated with a lower risk of type 2 diabetes. These associations remained significant in participants with a BMI of 25 or greater, an over 10-kg weight increase since age 20, and age younger than 75. Among participants with a BMI of 22 to 25, those whose waist circumference did not meet the metabolic syndrome criteria, and those who had not gained more than 10 kg since age 20, a weight increase of 3% or more was associated with the risk. In contrast, no associations were observed between weight change categories and the risk of type 2 diabetes in participants with a BMI of less than 22 or those aged 75 or older.

In the present study, a weight decrease of 1% or more was associated with a lower risk of type 2 diabetes in individuals with a BMI of 25 of greater and those who gained more than 10 kg since age 20, and a decrease of 3% or more was associated with a lower risk in those whose waist circumference met the metabolic syndrome criteria. These findings support the current Japan Society for the Study of Obesity guideline (8), based on previous studies showing that a weight loss of 3% or more over 6 months improved obesity-related parameters in individuals with obesity or metabolic syndrome (9). Importantly, this study also elucidated that a weight increase of 1% or more was associated with a higher type 2 diabetes risk, especially among individuals with a BMI of 25 or greater, those whose waist circumference met the metabolic syndrome criteria, and those who gained more than 10 kg since age 20. Moreover, a weight increase of 3% or more was associated with a higher risk even in those with a BMI of 22 to 25, those whose waist circumference did not meet the metabolic syndrome criteria, and those who had not gained more than 10 kg since age 20. These findings highlight the importance of weight management, including preventing weight increase, in lowering type 2 diabetes risk, not only for individuals with obesity but also for those with normal BMI, without abdominal obesity, or without long-term weight gain since early adulthood. Our findings further support the recommendation of the American Diabetes Association to screen all Asian American adults with a BMI of 23 or greater for diabetes (21).

In our study, weight change from age 20 was assessed by a single self-reported question asking whether participants had gained more than 10 kg since that age. This variable reflects cumulative long-term weight increase rather than short-term changes. While weight gain since age 20 has been associated with diabetes risk (22), there are no recommendations for weight management incorporating weight at age 20. Our findings suggest that weight management strategies may need to account for not only current BMI or waist circumference but also weight history from age 20.

In contrast, no associations were observed between each category of weight change and type 2 diabetes risk in individuals with a BMI less than 22. The Asian individuals with type 2 diabetes are characterized by a relatively low BMI onset (15), possibly because glucose intolerance in these individuals is caused by decreased insulin secretion rather than increased insulin resistance (23, 24). Our finding may support the evidence that type 2 diabetes in individuals with low BMI has distinct pathophysiological features.

The stratified analyses by age revealed associations between weight change and type 2 diabetes risk among individuals younger than 75 years, but not among those aged 75 or older. This discrepancy may reflect opposing mechanisms affecting glucose tolerance in older adults: Obesity causes insulin resistance, while weight loss due to sarcopenia contributes to reduced glucose tolerance (25-27). Given that Japan is a super-aged society, the Japanese Clinical Practice Guideline for Diabetes recommends adequate caloric intake and regular physical activity to prevent frailty (10). The findings of this study support these recommendations. It should be noted that because health examinations among people older than 75 are encouraged but not mandatory, diabetes incidence may be underreported in individuals who do not undergo health examinations regularly. This may lead to selection and detection biases and could partially explain the weaker association observed among individuals older than 75.

This is the first large prospective study in a Japanese population, including nonobese individuals, to examine the association between weight change and risk of type 2 diabetes and to clarify weight management goals according to BMI. The strengths of the present study include its prospective design, the large sample size in the general population, and the refined detection of type 2 diabetes using a comprehensive medical database that encompasses health examinations and health insurance claims. Importantly, while previous studies have predominantly focused on overweight or obese individuals and the benefits of weight loss, our study demonstrates that weight increase also confers an elevated risk of type 2 diabetes among individuals with normal BMI. This finding is particularly relevant in Asian populations, where a substantial proportion of cases occur among people at lower BMI levels. However, several limitations should be noted. First, the generalizability might be limited because the data from health examinations used in the present study were from individuals older than 40, and participants were mostly self-employed or nonregular employees, relatively older, and living in rural areas, which may not be representative of the overall Japanese population. Second, information on physical activity, walking speed, dietary habits, and weight change from age 20 was based on self-reported questionnaires, which may contain measurement errors. However, the questionnaires used in the present study were developed by the Ministry of Health, Labour and Welfare of Japan based on existing evidence and have been widely used in previous studies (28-30). In addition, the consistency of our results with other previous studies on weight loss and type 2 diabetes suggests that the observed associations were unlikely to be explained by measurement errors. Third, despite multivariable adjustment, residual confounding may remain. For example, factors such as muscle mass and socioeconomic status were not fully accounted for in this study. Fourth, we assessed weight changes over only 1 year in this study. Further investigation is needed to evaluate weight changes over longer periods.

In conclusion, a weight decrease of 1% or more over 1 year was associated with a lower risk of type 2 diabetes, particularly among individuals with a BMI of 25 or greater, those who had gained more than 10 kg since age 20, and those younger than 75. Moreover, a weight increase of 3% or more was associated with a higher risk even in those with a BMI of 22 to 25, those whose waist circumference did not meet the metabolic syndrome criteria, and those who had not gained more than 10 kg since age 20. Preventing weight gain, in addition to reducing weight, is important for the prevention of type 2 diabetes in Japanese individuals, particularly among those with a BMI of 22 or greater.

## Data Availability

The data that support the findings of this study are available from Kochi Prefecture, but restrictions apply to the availability of these data, which were used under license for the current study and, therefore, are not publicly available. Data are, however, available from the authors on reasonable request and with the permission of Kochi Prefecture.

## Abbreviations

BMI: body mass index
HbA_1c_: glycated hemoglobin A_1c_
HDL-C: high-density lipoprotein cholesterol
ICD10: International Statistical Classification of Diseases and Related Health Problems 10th Revision
KDB: Kokuho Database
LDL-C: low-density lipoprotein cholesterol
NGSP: national glycohemoglobin standardization program
